# The relationship between sexual activity and sexual attitudes among breast cancer survivors in China

**DOI:** 10.1002/cam4.2874

**Published:** 2020-03-24

**Authors:** Rui Yan, Jinming Yu, Tetsuya Tanimoto, Akihhiko Ozaki, Xinyuan Lu, Beibei Che, Yaxuan Zhang, Panzhen Chen, Jiwei Wang

**Affiliations:** ^1^ Institute of Clinical Epidemiology Key Laboratory of Public Health Safety Ministry of Education Key Lab of Health Technology Assessment of Ministry of Health School of Public Health Fudan University Shanghai China; ^2^ Medical Governance Research Institute Tokyo Japan; ^3^ Jyoban Hospital of Tokiwa Foundation Fukuhsima Japan

**Keywords:** breast cancer survivors, cross‐sectional studies, sexual activity, sexual attitude, sexual satisfaction

## Abstract

**Purpose:**

Less is known about sexual attitudes of breast cancer survivors (BCSs) and its association with sexual activity and sexual dissatisfaction.

**Methods:**

We investigated the proportion of sexual activity and sexual dissatisfaction in a cross‐sectional study among 341 Chinese BCSs aged 30‐75 years old, and we described their association with sexual attitudes, as well as socio‐demographic characteristics, physical health conditions, and mental health problems.

**Results:**

Only 83 (24.3%) individuals reported sexual activity in the past year. More than 50% of BCSs considered that sexual activity had adverse impact on their disease recovery. The sexual attitudes such as “sexual activity may impede disease recovery” (adjusted odds ratio [AOR] = 0.51; 95% confidence interval, 95% CI: 0.30‐0.88), “sexual activity may cause cancer recurrence or metastasizes” (AOR = 0.51; 95% CI: 0.30‐0.87), and “their partner fear contracting cancer by sexuality” (AOR = 0.47; 95% CI: 0.23‐0.98) were significantly associated with decreased likelihood of reporting sexual activity in the past year. Although 201 (58.9%) BCSs reported that breast cancer decreased the frequency of their sexual activity, only 37 (10.9%) had ever discussed sexuality with a doctor to seek advice.

**Conclusions:**

Most Chinese BCSs were sexually inactive. The sexual misconceptions about cancer were great barriers of sexual activity. Professional sexual education and consultation may be regarded as easy and effective intervention measures to correct BCSs' misguided sexual attitudes, and finally improving the overall sexual health for BCSs.

## Graphical abstract

Most Chinese BCSs were sexually inactive. The sexual misconceptions about cancer were great barriers of sexual activity. Professional sexual education and consultation may be regarded as easy and effective intervention measures to improve BCSs' sexual cognition, and finally improving the overall sexual health for BCSs.

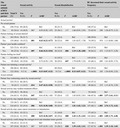

## 1 INTRODUCTION

Breast cancer (BC) is the most frequently diagnosed cancer and leading cause of cancer death for females worldwide.^1^ Thanks to early diagnosis and timely treatment, it is reported that 82.0% of the BC patients would live for at least 5 years in China,^2^ and thus BC is increasingly managed as a chronic illness.^3^ Return to normal life and experience best quality of life including sexuality has become active demand for BC survivor (BCS).

Sexuality contains sexual partnership, attitudes, activity, behavior, and function.^4^, ^5^ While sexuality is a key life component of health individuals, it is considered as a common “unmet needs” in BCSs.^6^The sexuality of BCSs could be greatly and negatively affected by BC and its treatment (eg, surgery, radiation therapy, chemotherapy, and endocrine therapy), the disturbances of body image, and mental health problems (eg, anxiety and depression).^7^, ^8^, ^9^ Sexually inactive may occur during BC treatment and extend into long‐term survivorship. However, little is known about the frequency of sexual activity in Chinese BCSs.

Sexual health is a state of physical, emotional, mental, and social well‐being in relation to sexuality.^10^ Sexual satisfaction is an important indicator of sexual health.^11^ Sexual satisfaction is the global subjective evaluation of individual's sexual health,^12^ and it involved both physical pleasure and emotional satisfaction.^13^ Sexual satisfaction of cancer patients are impaired compared with the general population,^14^ and individuals with sexual activity reported higher proportion of sexual satisfaction than those without sexual activity.^15^

Some sexual attitudes (eg, sexual activity is not safe for cancer patients, could weaken the effect of cancer treatment, and cause cancer recurrence)^16^ may also inhibit BCSs' sexual activity, yet is a neglected area. Estrogen levels is enhanced for female in sexual activity, and it have been proven that the carcinogenic effects of estrogens was involved in BC development.^17^ Scarcity of sexual knowledge and the fear of recurrence or metastasis may result in BCSs consider that sexual activity may change the estrogen level and stimulate tumor growth. However, as we known, sexuality is not a taboo for BCSs, and we found no research revealed that sexual activity was related to BC recurrence and metastasis. Yet sexual attitudes were not well described in BCSs and it is important to determine whether sexual attitudes was associated with BCSs’ sexual activity and sexual satisfaction.

Effective patient‐doctor communication about sexuality could correct the misguided sexual attitudes about cancer. However, a United States' survey of 261 gynecologic and BC patients shown that only 7% of women sought medical help for sexual issues.^18^ It was difficult for both patients and physicians to be open about sexual issues. Moreover, the sexual issues of cancer patients were always on the periphery of cancer care and often underestimated and neglected by the clinicians, due to personal embarrassment, having no time, or lack of communication skills and experience.^18^, ^19^

We designed a cross‐sectional study targeting the Chinese BCSs living in Shanghai who received comparable level of treatment with high‐income countries to provide data on multiple measures of sexuality: sexual activity, sexual satisfaction, and sexual attitudes. The primary hypothesize of our study was that the physicals health (general health, cancer‐related characteristics, comorbid chronic disease), mental health problems (anxiety and depression), and body image would remain associated with sexual activity and sexual dissatisfaction, after adjustment for age, marital status, time since cancer diagnosis, and self‐rated health. A second hypothesis was that the misguided sexual attitudes were associated with sexually inactive and sexual dissatisfaction.

## 2 MATERIALS AND METHODS

### 2.1 Study population

Shanghai Cancer Rehabilitation Club (SCRC) is a nongovernment and not‐for‐profit organization that serving cancer survivors exclusively. It contains 20 branches covered all the 17 districts in Shanghai, and recruited about 2000 cancer survivors every year from the local community and hospitals. From June to September 2018, we conducted a cross‐sectional study targeting all new female BC members that registered to SCRC in 2018.

Pathologically confirmed female BC patients aged 18 years or older who had completed their initial treatments were recruited in our study. Eligible participants also needed to be independently participated in the activities of SCRC, and have no cognitive impairment. All participants provided written informed consent after the detailed explanation of the study. Sexual health education provided for respondents after they completed their questionnaire. Those respondents suffered from sexual problems could consult to our specially trained investigators, or be advised to seek further professional clinic help. The study was approved by the Medical Research Ethics Committee of the School of Public Health, Fudan University (The international registry no. IRB00002408 and FWA00002399).

### 2.2 Survey instrument

We aimed to reveal sexual satisfaction and sexual activity among BCSs using a survey instrument. Participants were asked to complete a self‐reported structured questionnaire, including three components as below.

#### 2.2.1 Component one: basic characteristics and health conditions

Component one included basic characteristics such as age, marital status, educational attainment, income, and body mass index (BMI) (kg/m^2^). Respondents were also asked to rate their physical health using the five response options: “very bad,” “bad,” “fair,” “good,” or “very good.” Participants were also asked whether they had any of the following common comorbid chronic diseases (“yes” or “no”) that diagnosed by secondary or tertiary hospitals in China: hypertension, hyperlipidemia, hyperuricemia, diabetes mellitus, heart and cardiovascular diseases, stroke, respiratory diseases, digestive diseases, and musculoskeletal diseases. The cancer‐related medical information (time since cancer diagnosis, types of treatment received, breast reconstruction, recurrence, and metastasis) were self‐reported and disease stage were collected from medical records.

#### 2.2.2 Component two: anxiety, depression, and body image

Participants were subsequently asked to complete the Zung self‐rating anxiety scale (SAS)^20^ and the Zung self‐rating depression scale (SDS).^21^ Previous studies indicated that the reliability and validity of Chinese version SAS and SDS scale are acceptable (Cronbach's *α* = 0.782 for SAS, 0.777 for SDS). Self‐rating anxiety scale and SDS had been used as efficient tools for screening anxiety and depression among Chinese populations.^22^, ^23^ Individuals were deemed to have anxiety or depression if they had a Standard SAS score ≥ 50 or standard SDS score ≥ 50. The questionnaire contains four body image questions from the European Organization for Research and Treatment of Cancer Breast Cancer‐Specific Quality of Life Questionnaire (QLQ‐BR23): “Have you felt physically less attractive as a result of your disease or treatment?” “Have you been feeling less feminine as a result of your disease or treatment?” “Did you find it difficult to look at yourself naked?” “Have you been dissatisfied with your body?”^24^ Each question of the QLQ‐BR23 has four response categories: “not at all,” “a little,” “quite a bit,” and “very much”, and we defined “Yes” as either “a little,” “quite a bit,” or “very much.”

#### 2.2.3 Component three: sexuality

In our study, sexual activity is defined as any sexual contact (vaginal, anal, or oral intercourse, kissing, fondling, or petting) with another person.^5^, ^15^, ^16^ Individuals reported sexual activity in the past year were considered as “sexually active.”^5^ Component three included sexual questions that taking previous qualitative and quantitative researches as the reference basis.^5^, ^16^, ^18^, ^25^ Participants were asked to think about their sexuality in the past year and answer to following questions.

- Have you ever had any sexual activity (vaginal, anal, or oral intercourse, kissing, fondling, or petting) in the last year?^5^ (response options: “Yes” or “No”)
- During the past year, how satisfied have you been with your overall sex life ? (response options: “very satisfied” “moderately satisfied” “neither satisfied nor dissatisfied” “moderately dissatisfied” “very dissatisfied.”)
- How did the BC affect the frequency of your sexual activity? (response options: “obvious decreased” “decreased” “neither decreased nor increased” “increased” “obvious increased”)
- Since you were diagnosed with BC, have you ever discussed sexuality with a doctor to seek clinical advice for problems related to sexuality?^18^ (response options: “Yes” or “No”)

All the participants were asked whether they agree with several sexual attitudes related to BC. (eg, “My Partner did not initiate a sexual activity,” “My partner fear contracting cancer by sexual activity,” “Sexual activity may cause cancer recurrence or metastasize,” and “Sexual activity could change the estrogen level and stimulate tumor growth”)^16^, ^25^ (response options: “Yes” or “No”).

### 2.3 Statistical analysis

Chi‐square test or Fisher's exact test were used to examine the association between each sexual question and individuals' characteristics. Using logistic regression models, we estimated factors associated with engaging in sexual activity in the past year, reporting sexual dissatisfaction, and experiencing decreased sexual activity affected by BC. Age group (<55, 55‐64, and ≥65 years), marital status (married/cohabiting, and single/separated/divorced/widowed), self‐rated health status (very bad/bad, fair, and good/very good), and time since cancer diagnosis (<3, 3‐5, and ≥5 years) as covariates in light of the findings of previous studies,^14^, ^15^ and results were presented as odds ratio (OR) and its corresponding 95% confidence interval (95% CI). We also adjusted the number of comorbid chronic diseases (using categories of 0‐1, 2‐3, and ≥4) to investigate the association between each specific chronic health conditions and sexual activity taking account of the clustering of illnesses. Statistical analyses were performed with Statistical Analysis Software version 9.4, with a *P* < .05 indicating statistically significant.

## 3 RESULTS

A total of 532 BCSs met inclusion criteria and were invited to participate in our investigation. 22 individuals (4.1%) were excluded because they rejected to participate in our study, 150 respondents (28.2%) only finished the component one and component two, and refused to answer the sexual questions. Nineteen respondents (3.6%) were also excluded due to the large proportion of missing data in their questionnaires. At last, our final respondents comprised 341 (64.1%) BCSs with the mean age of 58 years (range 30‐75) (Table 1). Approximately 90% of them were married or cohabiting, and 88.6% BCSs were conducted with mastectomy.

**Table 1:** Reporting of sexual activity, sexual dissatisfaction, and BC decreased their sexual activity frequency in relation to demographic characteristics

Demographic characteristics	Total	Sexual activity			Sexual dissatisfaction			BC decreased their sexual activity frequency		
		N (%)	*P* a	AOR	N (%)	*P* a	AOR	N (%)	*P* a	AOR
All	341	83 (24.3)			81 (23.8)			201 (58.9)		
Age (y)										
Mean [SD](range)	58.64 [6.61] (30‐75)									
<55^b^	86 (25.2%)	38 (44.2)		Ref	25 (29.1)		Ref	57 (66.3)		Ref
55‐64	191 (56.0%)	35 (18.3)		**0.31 (0.17, 0.55)**	43 (22.5)		0.66 (0.36, 1.21)	116 (60.7)		0.73 (0.42, 1.26)
≥65	64 (18.8%)	10 (15.6)	**<.001**	**0.27 (0.12, 0.61)**	13 (20.3)	.382	0.57 (0.25, 1.28)	28 (43.8)	**.016**	**0.36 (0.18, 0.72)**
Marital status										
Married/cohabiting	308 (90.3%)	77 (25.0)		Ref	69 (22.4)		Ref	182 (59.1)		Ref
Single/separated/divorced/widowed	33 (9.7%)	6 (18.2)	.386	0.84 (0.32, 2.23)	12 (36.4)	.073	2.06 (0.94, 4.54)	19 (57.6)	.867	0.93 (0.44, 1.96)
Education										
<High school	198 (58.1%)	51 (25.8)		Ref	52 (26.3)		Ref	127 (64.1)		Ref
High school	118 (34.6%)	25 (21.2)		0.97 (0.54, 1.75)	21 (17.8)		0.59 (0.32, 1.07)	64 (54.2)		**0.61 (0.37, 0.99)**
>High school	25 (7.3%)	7 (28.0)	.596	1.01 (0.36, 2.79)	8 (32.0)	.139	1.73 (0.66, 4.53)	10 (40.0)	**.030**	**0.40 (0.17, 0.97)**
Household per capita income (yuan/mo)										
<2000	60 (17.6%)	17 (28.3)		Ref	13 (21.7)		Ref	38 (63.3)		Ref
~2000	161 (47.2%)	38 (23.6)		0.86 (0.42, 1.75)	42 (26.1)		1.19 (0.57, 2.50)	90 (55.9)		0.71 (0.38, 1.34)
≥4000	120 (35.2%)	28 (23.3)	.729	0.75 (0.36, 1.58)	26 (21.7)	.623	1.09 (0.51, 2.39)	73 (60.8)	.530	0.88 (0.45, 1.79)

Note

All models were adjusted for age, marital status, self‐reported general health status, and time since cancer diagnosis.

Bold face *P* < .05.

Abbreviations: AOR, adjusted odds ratio; BC, breast cancer.

a Chi‐square test or Fisher's exact test.

b The 86 individuals in <55 age group include 5 individuals <45 y and 81 individuals 45‐54 y.

### 3.1 Sexual activity in the past year

Among the participants, 83(24.3%) individuals reported having sexual activity in the past year. In bivariate analysis (Table 1), younger age, premenopausal, good/very good self‐reported health status, and those without diabetes mellitus were associated with high proportion in reporting sexual activity in the past year. (Tables 1, 2, 3) On the basis of the logistic regression analysis, after adjustment for marital status, self‐reported health status, and time since diagnosis, those aged 55‐64 years (adjusted odd ratio [AOR] = 0.31; 95% CI: 0.17‐0.55) and ≥65 years (AOR = 0.27; 95% CI: 0.12‐0.61) were significantly less likely to have sexual activity in the past year than younger BCSs (aged < 55 years) (Table 1). After adjustment for age, marital status, time since diagnosis, self‐rated health status, and number of comorbidity, diabetes mellitus were associated with a lower likelihood of engaging in sexual activity (AOR = 0.18; 95% CI: 0.04‐0.83) (Table 3).

**Table 2:** Reporting of sexual activity, sexual dissatisfaction, and BC decreased their sexual activity frequency in relation to general health and cancer‐related conditions

	Total	Sexual activity			Sexual dissatisfaction			BC decreased their sexual activity frequency		
		N (%)	*P* a	AOR	N (%)	*P* a	AOR	N (%)	*P* a	AOR
General Health										
BMI (kg/m^2^)										
Underweight (<18.5)	9 (2.6%)	1 (11.1)		Ref	4 (44.4)		Ref	7 (77.8)		
Normal weight (18.5‐24.9)	242 (71.0%)	55 (22.7)		3.08 (0.35, 27.31)	49 (20.3)		0.32 (0.07, 1.31)	132 (54.6)		0.41 (0.08, 2.06)
Overweight (25.0‐29.9)	79 (23.2%)	24 (30.4)		4.72 (0.51, 43.95)	25 (31.7)		0.63 (0.14, 2.76)	55 (69.6)		0.80 (0.15, 4.26)
Obesity (≥30)	11 (3.3%)	3 (27.3)	.421	2.83 (0.22, 37.14)	3 (27.3)	.087	0.47 (0.07, 3.23)	7 (63.6)	.07	0.56 (0.07, 4.21)
Menopausal status^b^										
Regular menstrual	10 (3.7%)	4 (40.0)		Ref	0 (0)		Ref	8 (66.7)		Ref
Irregular menstrual	20 (7.4%)	10 (50.0)		0.89 (0.17, 4.65)	6 (30)		—	16 (76.2)		1.20 (0.20, 7.30)
Postmenopausal	242 (89.0%)	56 (23.1)	**.018**	0.54 (0.14, 2.16)	61 (25.2)	.164	—	177 (57.5)	.207	0.59 (0.14, 2.46)
Self‐reported general health status										
Bad/very bad	32 (9.4%)	4 (12.5)		Ref	13 (40.6)		Ref	21 (65.6)		Ref
Fair	206 (60.6%)	42 (20.3)		1.68 (0.54, 5.16)	45 (21.7)		**0.39 (0.17, 0.87)**	125 (60.4)		0.75 (0.34, 1.68)
Good/very good	102 (30.0%)	37 (36.3)	**.002**	**3.28 (1.03, 10.42)**	23 (22.6)	.061	**0.34 (0.17, 0.83)**	55 (53.9)	.400	0.51 (0.21, 1.19)
Cancer‐related characteristics										
Stage^c^										
0‐I	115 (37.8%)	24 (20.9)		Ref	25 (21.7)		Ref	64 (55.7)		Ref
II	140 (46.1%)	43 (30.7)		1.54 (0.84, 2.82)	39 (27.9)		1.39 (0.77, 2.52)	90 (64.3)		1.32 (0.79, 2.21)
III‐IV	49 (16.1%)	9 (18.4)	.098	0.78 (0.32, 1.90)	9 (18.4)	.314	0.78 (0.33, 1.86)	27 (55.1)	.297	0.93 (0.47, 1.84)
Time since diagnosis (y)^c^										
<3	115 (33.7%)	30 (26.1)			25 (21.7)			72 (62.6)		
~3	132 (38.7%)	30 (22.7)		0.79 (0.43, 1.47)	41 (31.1)		1.71 (0.94, 3.09)	76 (57.6)		0.88 (0.52, 1.49)
≥5	94 (27.6%)	23 (24.5)	.828	1.19 (0.61, 2.23)	15 (16.0)	**.026**	0.67 (0.32, 1.40)	53 (56.4)	.608	0.85 (0.48, 1.52)
Surgery type										
Lumpectomy	39 (11.4%)	14 (35.9)		Ref	7 (18.0)		Ref	20 (51.3)		Ref
Mastectomy	302 (88.6%)	69 (22.9)	.074	0.51 (0.24, 1.11)	74 (24.5)	.365	1.89 (0.78, 4.57)	181 (59.9)	.301	1.49 (0.75, 2.94)
Breast reconstruction^e^										
Yes	8 (2.7%)	4 (50.0)		Ref	2 (25.0)		Ref	5 (62.5)		Ref
No	294 (97.4%)	65 (22.2)	.081	0.38 (0.09, 1.70)	72 (24.9)	.316	0.98 (0.19, 5.20)	176 (59.9)	.999	1.06 (0.25, 4.60)
Radiotherapy										
No	225 (66.0%)	55 (24.4)		Ref	56 (24.9)		Ref	129 (57.3)		Ref
Yes	116 (34.0%)	28 (24.1)	.95	0.81 (0.46, 1.40)	25 (21.6)	.492	0.77 (0.44, 1.34)	72 (62.1)	.399	1.14 (0.72, 1.83)
Chemotherapy										
No	51 (15.0%)	11 (21.6)		Ref	12 (23.5)		Ref	23 (45.1)		Ref
Yes	290 (85.0%)	72 (24.8)	.617	1.09 (0.52, 2.32)	69 (23.8)	.967	1.08 (0.53, 2.22)	178 (61.4)	**.029**	**1.88 (1.02, 3.48)**
Hormonal therapy										
Never	125 (36.7%)	25 (20)		Ref	32 (25.6)		Ref	72 (57.6)		Ref
Ever	33 (9.7%)	9 (27.3)		1.68 (0.66, 4.32)	7 (21.2)		1.00 (0.38, 2.65)	17 (51.5)		0.82 (0.37, 1.82)
Current	183 (53.6%)	49 (26.8)	.364	1.44 (0.81, 2.54)	42 (23.0)	.811	0.81 (0.47, 1.39)	112 (61.2)	.54	1.13 (0.70, 1.81)
Recurrance										
No	331 (97.1%)	82 (24.8)		Ref	80 (24.2)		Ref	196 (59.2)		Ref
Yes	10 (2.9%)	1 (10.0)	.284	0.33 (0.04, 2.79)	1 (10.0)	.299	0.36 (0.04, 3.03)	5 (50.0)	.56	0.78 (0.21, 2.84)
Metastasis										
No	323 (94.7%)	79 (24.5)		Ref	76 (23.5)		Ref	188 (58.2)		Ref
Yes	18 (5.3%)	4 (22.2)	.83	0.71 (0.21, 2.35)	5 (27.8)	.68	1.24 (0.41, 3.77)	13 (72.2)	.239	2.15 (0.72, 6.42)

Note

All models were adjusted for age, marital status, self‐reported general health status, and time since cancer diagnosis.

Bold face *P* < .05.

Abbreviations: AOR, adjusted odds ratio; BC, breast cancer.

a Chi‐square test or Fisher's exact test.

b Analysis restricted to individuals aged 45‐64 y (n = 272). BC deemed to be postmenopausal when they had not menstruated in the past year.

Cancer stage was unknown for 37 subjects. Stage 0‐Ⅰ groups include 10 subjects with stage 0 and 105 subjects with stage Ⅰ. Stage Ⅲ‐Ⅳ groups include 44 subjects with stage Ⅲ and 5 subjects with stage Ⅳ.

c Subjucts who received their cancer diagnosis <3 y (n = 115) includes 2 subjects with cancer diagnosis <1 y and 113 subjects with cancer diagnosis 1‐3 y.

Analysis restricted to individuals with mastectomy (n = 302).

**Table 3:** Reporting of sexual activity, sexual dissatisfaction, and BC decreased their sexual activity frequency in relation to comorbid chronic diseases

Comorbidity	Total	Sexual activity			Sexual dissatisfaction			BC decreased their sexual activity frequency		
		N (%)	*P* a	AOR	N (%)	*P* a	AOR	N (%)	*P* a	AOR
Hypertension										
No	247 (72.4%)	66 (26.7)		Ref	59 (23.9)		Ref	142 (57.5)		Ref
Yes	94 (27.6%)	17 (18.1)	.097	0.59 (0.29, 1.17)	22 (23.4)	.926	1.23 (0.62, 2.43)	59 (62.8)	.376	1.46 (0.83, 2.58)
Hyperlipidemia										
No	239 (70.1%)	63 (26.4)		Ref	61 (25.5)		Ref	143 (59.8)		Ref
Yes	102 (29.9%)	20 (19.6)	.183	0.71 (0.35, 1.43)	20 (19.6)	.24	0.71 (0.35, 1.45)	58 (56.9)	.61	0.88 (0.49, 1.56)
Hyperuricemia										
No	325 (95.3%)	82 (25.2)		Ref	75 (23.1)		Ref	193 (59.4)		Ref
Yes	16 (4.7%)	1 (6.3)	.084	0.21 (0.02, 1.71)	6 (37.5)	.186	1.97 (0.61, 6.42)	8 (50.0)	.456	0.65 (0.22, 1.95)
Diabetes mellitus										
No	307 (90.0%)	81 (26.4)		Ref	68 (22.2)		Ref	182 (59.3)		Ref
Yes	34 (10.0%)	2 (5.9)	**.008**	**0.18 (0.04, 0.83)**	13 (38.2)	**.036**	**2.92 (1.25, 6.79)**	19 (55.9)	.702	0.90 (0.41, 1.95)
Heart and cardiovascular										
No	298 (87.4%)	71 (23.8)		Ref	69 (23.2)		Ref	171 (57.4)		Ref
Yes	43 (12.6%)	12 (27.9)	.559	1.70 (0.75, 3.88)	12 (27.9)	.494	1.61 (0.72, 3.63)	30 (69.8)	.123	2.05 (0.96, 4.39)
Stroke										
No	334 (98.0%)	81 (24.3)		Ref	78 (23.4)		Ref	196 (58.7)		Ref
Yes	7 (2.1%)	2 (28.6)	.792	1.84 (0.33, 10.12)	3 (42.9)	.23	3.18 (0.65, 15.58)	5 (71.4)	.497	1.75 (0.32, 9.48)
Respiratory diseases										
No	309 (90.6%)	74 (24.0)		Ref	76 (24.6)		Ref	182 (58.9)		Ref
Yes	32 (9.4%)	9 (28.1)	.6	1.47 (0.59, 3.66)	5 (15.6)	.256	0.54 (0.19, 1.54)	19 (59.4)	.958	1.08 (0.49, 2.41)
Digestive diseases										
No	186 (54.6%)	43 (23.1)		Ref	49 (26.3)		Ref	115 (61.8)		Ref
Yes	155 (45.5%)	40 (25.8)	.565	1.20 (0.61, 2.40)	32 (20.7)	.218	0.88 (0.43, 1.78)	86 (55.5)	.236	0.67 (0.37, 1.21)
Musculoskeletal diseases										
No	291 (85.3%)	71 (24.4)		Ref	66 (22.7)		Ref	175 (60.1)		Ref
Yes	50 (14.7%)	12 (24.0)	.952	1.23 (0.55,2.79)	15 (30.0)	.261	1.81 (0.83,3.93)	26 (52.0)	.279	0.71 (0.36,1.41)
Number of comorbidity										
0‐1	184 (53.9%)	53 (28.8)		Ref	41 (22.3)		Ref	112 (60.9)		Ref
2‐3	125 (36.7%)	24 (19.2)		0.83 (0.45, 1.53)	31 (24.8)		1.17 (0.65, 2.11)	71 (56.8)		0.93 (0.57, 1.54)
≥4	32 (9.4%)	6 (18.8)	.115	0.91 (0.33, 2.47)	9 (28.1)	.729	1.56 (0.64, 3.85)	18 (56.3)	.735	1.05 (0.47, 2.33)

Note

All models were adjusted for age, marital status, self‐reported general health status, and time since cancer diagnosis.

Models investigating each specific comorbid chronic disease were also adjusted for the number of comorbidity.

Bold face *P* < .05.

Abbreviations: AOR, adjusted odds ratio; BC, breast cancer.

a Chi‐square test or Fisher's exact test.

### 3.2 Sexual dissatisfaction

Among the participants, 81(23.8%) reported being dissatisfied with their sexuality. In bivariate analysis (Tables 1 and 2), only time since diagnosis was associated significantly with the likelihood of reporting sexual dissatisfaction. After adjustment for age, marital status, and time since diagnosis, fair self‐reported health status (AOR = 0.39; 95% CI: 0.17‐0.87) and good/very good self‐reported health status (AOR = 0.34; 95% CI: 0.17‐0.83) were associated with lower likelihood of sexual dissatisfaction than those with bad/very bad self‐reported health (Table 2).

For common comorbid chronic diseases, the higher proportion of sexual dissatisfaction was significant only for those with diabetes mellitus (AOR = 2.92; 95% CI: 1.25‐6.79) (Table 3). BCSs that felt physically less attractive as a result of their disease or treatment (AOR = 2.06; 95% CI: 1.17‐3.65) and feeling less feminine as a result of their disease or treatment (AOR = 1.78; 95% CI: 1.02‐3.13) were associated significantly with the higher likelihood of reporting sexual dissatisfaction (Table 4).

**Table 4:** Reporting of sexual activity, sexual dissatisfaction, and BC decreased their sexual activity frequency in relation to psychologic variables and body image

Psychologic variables	Total	Sexual activity			Sexual dissatisfaction			BC decreased their sexual activity frequency		
		N (%)	*P* a	AOR	N (%)	*P* a	AOR	N (%)	*P* a	AOR
Anxiety										
No	237 (69.5%)	57 (24.1)			55 (23.2)			135 (57.0)		
Yes	104 (30.5%)	26 (25.0)	.851	1.25 (0.71, 2.22)	26 (25.0)	.72	1.06 (0.61, 1.87)	66 (63.5)	.261	1.29 (0.79, 2.11)
Depression										
No	196 (57.5%)	49 (25.0)			41 (20.9)			109 (55.6)		
Yes	145 (42.5%)	34 (23.5)	.741	1.00 (0.59, 1.72)	40 (27.6)	.153	1.39 (0.82, 2.35)	92 (63.5)	.146	1.28 (0.81, 2.02)
Felt physically less attractive as a result of your disease or treatment										
No	142 (41.6%)	34 (23.9)			24 (16.9)			72 (50.7)		
Yes	199 (58.4%)	49 (24.6)	.885	1.31 (0.75, 2.28)	57 (28.6)	**.012**	**2.06 (1.17, 3.65)**	129 (64.8)	**.009**	**1.73 (1.09, 2.75)**
Find it difficult to look at yourself naked										
No	122 (35.8%)	35 (28.7)			28 (23.0)			66 (54.1)		
Yes	219 (64.2%)	48 (21.9)	.163	0.82 (0.47, 1.41)	53 (24.2)	.795	1.01 (0.58, 1.73)	135 (61.6)	.175	1.29 (0.81, 2.05)
Feeling less feminine as a result of your disease or treatment										
No	140 (41.1%)	35 (25.0)			25 (17.9)			65 (46.4)		
Yes	201 (58.9%)	48 (23.9)	.813	1.02 (0.59, 1.75)	56 (27.9)	**.033**	**1.78 (1.02, 3.13)**	136 (67.7)	**<.001**	**2.34 (1.47, 3.72)**
Dissatisfied with your body										
No	150 (44.0%)	40 (26.7)			30 (20.0)			78 (52.0)		
Yes	191 (56.0%)	43 (22.5)	.375	1.05 (0.60, 1.82)	51 (26.7)	.149	1.40 (0.80, 2.46)	123 (64.4)	**.021**	**1.62 (1.02, 2.58)**

Note

All models were adjusted for age, marital status, self‐reported general health status, and time since cancer diagnosis. Bold face *P* < .05.

Abbreviations: AOR, adjusted odds ratio; BC, breast cancer.

a Chi‐square test or Fisher's exact test.

### 3.3 Decreased sexual activity frequency

Among the participants, 201(58.9%) individuals reported that the frequency of their sexual activity was decreased due to BC. (Table 1) BCSs aged ≥65 years (AOR = 0.36; 95% CI: 0.18‐0.72), and with higher levels of education were significantly less likely to consider BC as the cause of decreased frequency of sexual activity (Table 1). Chemotherapy (AOR = 1.88; 95% CI: 1.02‐3.48), feeling physically less attractive as a result of their disease or treatment (AOR = 1.73; 95% CI: 1.09‐2.75), feeling less feminine as a result of their disease or treatment (AOR = 2.34; 95% CI: 1.47‐3.72) and been dissatisfied with your body (AOR = 1.62; 95% CI: 1.02‐2.58) were associated significantly with increased likelihood of reporting BC decreased their sexual activity frequency (Table 4).

### 3.4 Sexual partner

The lack of partner’s sexual interest (AOR = 0.42; 95% CI: 0.23‐0.77) was associated with sexually inactivity. (Table 5) Partner's poor health condition and partner not initiating sexual activity were associated with both BCSs’ sexually inactivity and sexual dissatisfaction.

**Table 5:** Reporting of sexual activity, sexual dissatisfaction, and BC decreased their sexual activity frequency in relation to sexual partner and individual's sexual attitude

The sexual attitude of BCS and their partner	Total N (%)	Sexual activity			Sexual dissatisfaction			BC decreased their sexual activity frequency		
		Yes N (%)	*P*	AOR^a^	Yes N (%)	*P*	AOR^a^	Yes N (%)	*P*	AOR^a^
*Sexual partner*										
Without sexual partner										
No	258 (75.6)	69 (26.9)		Ref	56 (21.7)		Ref	148 (57.4)		Ref
Yes	83 (24.4)	14 (16.9)	.067	0.55 (0.28, 1.07)	25 (30.1)	.117	1.66 (0.93, 2.95)	53 (63.9)	.296	1.26 (0.75, 2.12)
Partner lacking of sexual interest^b^										
No	183 (59.4)	58 (31.7)		Ref	41 (22.4)		Ref	108 (59.0)		Ref
Yes	125 (40.6)	19 (15.2)	**.001**	**0.42 (0.23, 0.77)**	28 (22.4)	.999	1.01 (0.57, 1.78)	74 (59.2)	.974	1.11 (0.69, 1.80)
Partner' poor health condition^b^										
No	223 (72.4)	65 (29.2)		Ref	41 (18.4)		Ref	129 (57.9)		Ref
Yes	85 (27.6)	12 (14.1)	**.007**	**0.46 (0.23, 0.94)**	28 (32.9)	**.006**	**2.19 (1.20, 4.00)**	58 (64.2)	.472	1.33 (0.77, 2.29)
Partner feeling too tired^b^										
No	213 (69.1)	59 (27.7)		Ref	41 (19.3)		Ref	119 (55.9)		Ref
Yes	95 (30.8)	18 (19.0)	.101	0.72 (0.39, 1.35)	28 (29.5)	**.047**	1.79 (0.99, 3.22)	63 (66.3)	.085	1.61 (0.95, 2.72)
Partner not initiating a sexual activity^b^										
No	186 (60.4)	68 (36.6)		Ref	68 (36.6)		Ref	103 (55.4)		Ref
Yes	122 (39.6)	9 (7.4)	**<.0001**	**0.15 (0.07, 0.32)**	9 (7.4)	**<.0001**	**0.15 (0.07, 0.32)**	79 (64.8)	.102	1.54 (0.94, 2.52)
*Sexual attitude*										
Partner fear contracting cancer by sexual activity^b^										
No	232 (75.3)	66 (28.5)		Ref	51 (22.0)		Ref	133 (57.3)		Ref
Yes	76 (24.7)	11 (14.5)	**.014**	**0.47 (0.23, 0.98)**	18 (23.7)	.758	1.11 (0.59, 2.08)	49 (64.5)	.272	1.31 (0.76, 2.29)
Sexual activity may weaken treatment effects										
No	125 (36.7)	38 (30.4)		Ref	24 (19.2)		Ref	55 (44.0)		Ref
Yes	216 (63.3)	45 (20.8)	**.047**	0.61 (0.36, 1.05)	57 (26.4)	.132	1.65 (0.93, 2.91)	146 (67.6)	**<.001**	**2.57 (1.61, 4.11)** ^c^
Sexual activity may impede disease recovery										
No	125 (36.7)	41 (32.8)		Ref	23 (18.4)		Ref	54 (43.2)		Ref
Yes	216 (63.3)	42 (19.4)	**.006**	**0.51 (0.30, 0.88)**	58 (26.9)	.077	**1.81 (1.02, 3.22)**	147 (68.1)	**<.001**	**2.76 (1.72, 4.42)**
Sexual activity may cause cancer recurrence or metastasizes										
No	131 (38.4)	42 (32.1)		Ref	21 (16.0)		Ref	57 (45.3)		Ref
Yes	210 (61.6)	41 (19.5)	**.008**	**0.52 (0.30, 0.87)**	60 (28.6)	**.008**	**2.45 (1.35, 4.45)**	144 (68.6)	**<.001**	**2.80 (1.75, 4.48)**
Sexual activity could change the estrogen level and stimulate tumor growth										
No	154 (45.2)	43 (31.5)		Ref	28 (18.2)		Ref	74 (48.1)		Ref
Yes	187 (54.8)	50 (26.7)	.807	0.97 (0.59, 1.42)	53 (28.3)	**.028**	**1.59 (1.03, 2.41)**	127 (67.9)	**<.001**	**1.39 (1.18, 1.69)**

Note

Bold face *P* < .05.

a AOR: adjusted odds ratio, adjusted for age, marital status, self‐reported general health status, and time since cancer diagnosis.

b Analysis restricted to individuals married/cohabiting (n = 308).

### 3.5 Sexual attitudes and seeking behavior for clinic advice about sexual problems

Among the married/cohabiting individuals, 24.7% BCSs reported that they think their partner fears to be contracted with cancer by sexual activity, and this was associated with sexually inactivity (AOR = 0.47; 95% CI: 0.23‐0.98). For BCSs, the most commonly reported misguided sexual attitudes included “Sexual activity may weaken treatment effects” (63.2%), “Sex may impede disease recovery” (63.2%), “Sexual activity may cause cancer recurrence or metastasis” (61.5%), “Sexual activity could change the estrogen level and stimulate tumor growth” (54.7%).

After adjustment for age, marital status, time since diagnosis, and self‐reported health status, “sexual activity may impede disease recovery” (AOR = 0.51; 95% CI: 0.30‐0.88), and “sexual activity may cause cancer recurrence or metastasis” (AOR = 0.51; 95% CI: 0.30‐0.87) were associated significantly with sexual inactivity, sexual dissatisfaction, and an idea that BC decreased the frequency of their sexual activity (Table 5). Only 37(10.9%) reported having ever discussed sex with a doctor to seek clinic advice for sexual problems (data not shown).

## 4 DISCUSSION

As far as we known, this is the first study among Chinese BCSs that analyzed the sexuality from multiple dimensions benefited from the comprehensive analysis of sexuality including physical, psychological, and attitude. Our study shown that less than a quarter of BCSs (24.3%) reported that they had sexual activity during the past year in Shanghai, China, where style of living is modernized comparable to high‐income countries. As predicted, older age, poorer physical health, and postmenopausal status were associated with sexual inactivity. We have also identified strong associations between misguided sexual attitudes related to cancer, their partner’ not initiative and sexual inactivity in the past year.

Comorbid chronic disease, any additional clinical entity that coexists with cancer, was common in cancer survivors.^26^ We noted strong associations between less likelihood of sexual activity and higher likelihood of sexual dissatisfaction only for combined with diabetes mellitus, not for other specific chronic health conditions. Although few data were reported about the effects of diabetes mellitus medications on female sexuality, diabetes mellitus was generally associated with depression, and marital conflict,^27^ and women with diabetes mellitus were less likely to be sexually active than women without diabetes mellitus.^5^, ^28^

Breast cancer treatment may case various physical and functional changes to a woman's body, such as hair loss, scarring, and loss of (or part of) a breast and lymphedema. The BC related body image changes may make them felt less comfortable with their bodies, and may impact on their sexual desire and sexual function. In our study, BCSs who felt physically less attractive or less feminine reported more likely that BC decreased frequency of their sexual activity and sexual dissatisfaction. Breast is considered as a symbol of femininity and sexuality for women. Breast‐conserving surgery is the preferred surgical approach for early BC patients.^29^, ^30^ However, the proportion of breast‐conserving is quite low in China. The proportion of mastectomy in our study was obviously higher than that in other countries.^31^, ^32^ In the United States, breast reconstruction is considered as a part of the standard of care for BC patients treated with mastectomy^33^; however, only 2.7% BCSs received breast reconstruction after mastectomy in our study. The scarring or mastectomy that result from surgery can cause various psychosocial problems, including less feeling of attractiveness and satisfaction with body, reduced self‐esteem and self‐efficacy, and would have adverse impact on sexual experience.

Sexuality after BC should not be a taboo. However, at least a half BCSs held the misconception that sexual activity could change the estrogen level and stimulate tumor growth, weaken treatment effects, and result in cancer recurrence or metastasis, indicating that sexual misconception related to cancer was a great barrier of sexual activity. A nationwide survey of Japanese breast surgeons concerning sexuality‐related consultations in 2001 indicated that the most frequently consulted topic was related to the safety of having sex.^34^ Professional sexual consultation or education to raise awareness the sexual safety would be helpful to improve sexual health among BCSs. However, in China, the cancer management is more focused on cancer survival, recurrence, and metastasis. Sexual health is always on the periphery of cancer care, and frequently overlooked or ignored.

The improvement of sexual attitudes related to cancer needs the cooperation of patients and doctors. Physicians were encouraged to query patients’ sexual history as part of the routine assessment of new patients,^7^ and it is appropriate for individuals to initiatively discuss sexual issues after cancer with physicians.^18^ Yet very few (11%) BCSs had sought clinic advice about sexuality in our study. Reasons of patients' lack of initiative included unwillingness and the limited time spent facing the patient during the medical visit. As a personal privacy, patients may be reluctant or ashamed to discuss sexuality with doctors. Wang et al found that 70.6% of Chinese BCSs had actively sought sexual helps or information. However, their sexual knowledge mainly came from other BCSs (57.2%), friends (44.4%) and Internet (30.0%), rather than from doctors (6.1%).^25^ The limited face‐to‐face visit time with doctors may also make patients more willing to consult questions about survival, rather than sexuality. The poor communication was also associated with physicians' poor awareness and clinical skills. A previous study found that only 25% doctors and 20% nurses have ever discussed sexual issues with ovarian cancer patients, and “out of my responsibility” “embarrassment” were the most common reasons.^35^ A nationwide survey in Japan indicated that breast surgeons who agreed that “it was my responsibility to deal with BCSs’ sexual issues” were more likely to be consulted than those with the opinion that “if BCSs have any sexual issues, they will raise the topic before I ask” 34 Skills to make patients open the sexual topic is also important. Physicians should inform BCSs that there may exist some sexual side effects during or after cancer treatment, and questions like “What changes in sexuality have you noticed?” may be helpful to give BCS permission to talk about sex.^36^ If it is really embarrassing and hard to open the topic, patients still may benefit from an educational pamphlet distributed (preferably for free) to them.^19^

Individuals' sexual function and sexual satisfaction was strongly associated with their partner's sexual function, and this interaction may be multifactorial and complex.^37^ However, we did not collect information about the partner's sexual function, which may be a main limitation of this study. Additionally, partner's care and support can also play an important role in sexual health. A previous study showed that partner initiation of sex predicted greater marital satisfaction for BC patients.^38^ Partners should give BCSs more care and understanding, and pay attention to BCSs' emotional health and sexual demand. It is also OK to let the partner know what the BCSs thinks, and an open conversation with partner about the BCSs’ sexual desire can help to engage in sexual activity. In our study, 25.7% BCSs reported that their partners fear to be contracted with cancer by sexuality. Developing sexual education and popularizing that sexuality is safe and couldn't transmit cancer, could be helpful to correct partner's wrong sexual attitudes and improve BCSs’ sexual health.

The study has several limitations. First, participants were all members registered in SCRC susceptible to participation biases; for example, poor health may have affected the willingness to participate in our study. Second, most of the data in our study were self­reported, and we couldn't check the accuracy of the information we collected. Third, we did not collect information that whether BCSs' comorbid chronic disease were diagnosed before or after the cancer diagnosis, which treatment was adopted, and how well the comorbid chronic disease was controlled. Additionally, we did not collect information about the pathological type of BC, and it was necessary to explore its association with sexual health in further research. Fourth, the sample size in current study is not large. The individuals in some subgroup after stratified by various factors was limited, which may result in our analyses lacking of statistical power. The enrolled BCSs in our study also were unbalanced in the features of cancer stage, and most of them had an early stage of BC. Further studied with larger sample size should enroll more BCSs with advanced stage to validate our results. Fifth, the role of partner's sexual function was also strongly associated with individuals’ sexual activity. However, we did not collect information about the partner's sexual function, which may be a main limitation of this study. Finally, we only provided a possible association between sexual attitudes and sexual activity due to the cross‐sectional nature. Further studied should be conducted to identify the role of cognitive therapy on enhancing BCSs' sexual health.

In conclusion, our results demonstrate that most Chinese BCSs were sexually inactive. Sexual misconceptions, for example, sexual activity may cause cancer recurrence or metastasizes, were closely associated with sexually inactive. BCSs infrequently discussed sexual issues with physicians. The sexual care is scarce in Chinese cancer management. The professional sexual education and consultation may be regarded as easy and effective intervention measures to improve BCS' sexual knowledge. Chinese BCSs may benefit from the communication with healthcare professionals, even just one sentence to tell BCSs that sex is safe, or an educational pamphlet.

## CONFLICT OF INTEREST

The authors declare that they have no conflict of interest.

## RESEARCH INVOLVING HUMAN PARTICIPANTS

The study was approved by the Medical Research Ethics Committee of the school of public health, Fudan University (The international registry no. IRB00002408 and FWA00002399).

## INFORMED CONSENT

Informed consent was obtained from all individual participants included in the study.

## Data Availability

The data sets generated during and/or analyzed during the current study are available from the corresponding author on reasonable request.
